# Rapid phenotyping of knockout mice to identify genetic determinants of bone strength

**DOI:** 10.1530/JOE-16-0258

**Published:** 2016-10-01

**Authors:** Bernard Freudenthal, John Logan, Sanger Institute Mouse Pipelines, Peter I Croucher, Graham R Williams, J H Duncan Bassett

**Affiliations:** 1 Molecular Endocrinology LaboratoryDepartment of Medicine, Imperial College London, London, UK; 2 Mouse PipelinesWellcome Trust Sanger Institute, Wellcome Trust Genome Campus, Hinxton, Cambridge, UK; 3 Garvan Institute of Medical ResearchSydney, New South Wales, Australia

**Keywords:** osteoporosis, bone, genetics, gene discovery

## Abstract

The genetic determinants of osteoporosis remain poorly understood, and there is a large unmet need for new treatments in our ageing society. Thus, new approaches for gene discovery in skeletal disease are required to complement the current genome-wide association studies in human populations. The International Knockout Mouse Consortium (IKMC) and the International Mouse Phenotyping Consortium (IMPC) provide such an opportunity. The IKMC generates knockout mice representing each of the known protein-coding genes in C57BL/6 mice and, as part of the IMPC initiative, the Origins of Bone and Cartilage Disease project identifies mutants with significant outlier skeletal phenotypes. This initiative will add value to data from large human cohorts and provide a new understanding of bone and cartilage pathophysiology, ultimately leading to the identification of novel drug targets for the treatment of skeletal disease.

## Introduction

### A novel strategy for osteoporosis gene discovery

Studies of human monogenic extreme phenotype disorders have been instrumental in discovering genetic and molecular mechanisms of common diseases including obesity and diabetes (Yamagata *et al*. 1996, Montague *et al*. 1997). However, collection of human extreme phenotype cohorts takes many years and requires significant effort and financial resource. A new approach to osteoporosis gene discovery involves systematic identification of extreme skeletal phenotypes in mutant mouse lines that carry single-gene knockouts representing all the known protein-coding genes. This approach has been made possible by the International Knockout Mouse Consortium (IKMC), whose aim is to disrupt each of the protein-coding genes in C57BL/6 mice, and the International Mouse Phenotyping Consortium (IMPC) that has established a multidisciplinary and broad primary phenotype screen to characterise these mutant mice. By using samples from mice that have undergone the IMPC phenotyping pipeline, a bespoke rapid-throughput multi-parameter skeletal phenotyping platform has been applied systematically to detect significant phenotypes by screening minimal number of samples. This phenotyping programme exploits the excellent replication of human skeletal disease in mice, and novel susceptibility genes can be validated by interrogating human osteoporosis cohorts.

### ‘Known unknowns’ in osteoporosis

Osteoporosis is a worldwide healthcare problem that causes up to 9 million fractures annually (Johnell & Kanis 2006). Within the EU, it is estimated that osteoporosis affects 30 million people and osteoporotic fractures cost €37 billion annually (Hernlund *et al*. 2013). These numbers are projected to rise with the increasing elderly population. Osteoporotic hip fractures are associated with a significant rise in mortality (Sattui & Saag 2014), especially during the year following fracture when it is estimated to be 8–36% (Abrahamsen *et al*. 2009).

The most important risk factors for osteoporotic fracture are low bone mineral density (BMD) (clinically assessed by dual-energy X-ray absorptiometry (DEXA or DXA)), increasing age and history of fracture (Johnell *et al*. 2005). There are two key determinants of adult BMD: the peak bone mass attained in early adulthood and the rate of bone loss during ageing. Variation in BMD has a large heritable genetic component. This is known from observations of familial clustering of osteoporosis (Seeman *et al*. 1989, Keen *et al*. 1999) and from twin studies that have calculated that the heritable contribution lies between 40 and 90% (Mitchell & Yerges-Armstrong 2011). The heritable contribution to variance in BMD is greatest in early adulthood (Gueguen *et al*. 1995), yet variation in the rate of bone loss *per se* is also genetically determined (Kelly *et al*. 1993). Thus, genetic mechanisms contribute significantly to the risk of osteoporosis. Nevertheless, the known BMD-associated genetic variants account for only 5.8% of the total variance (Estrada *et al*. 2012), indicating that the majority of susceptibility genes have yet to be identified.

### Gene discovery from skeletal extreme phenotypes

Intrinsic and extrinsic factors, systemic hormones, neuronal innovation and mineral homeostasis can all affect bone mass. Significantly, many of the genes and signalling pathways involved in the intrinsic regulation of bone turnover and bone mass have been identified by the study of human monogenic disorders associated with extremes of BMD (Table 1). In traditional gene discovery, the loci of causative alleles would be identified by linkage analysis in the families of index cases, followed by positional cloning of the relevant genes (Alonso & Ralston 2014). Such studies have identified the two key regulatory pathways – canonical Wnt signalling and receptor activator of nuclear factor kappa-B ligand (RANKL)/RANK/osteoprotegerin (OPG) that respectively regulate the function of osteoblasts and osteoclasts. Both these pathways have subsequently been targeted by novel osteoporosis treatments.

**Table 1:** Monogenic disorders that have identified key skeletal genes in bone remodelling.

	**Disease**	**Clinical features**	**Gene**	**Mechanism**	**Reference**
Reduced bone mass	Osteoporosis- pseudoglioma syndrome (OPPGS)	Reduced bone mass and blindness	*LRP5*	Loss-of-function mutations disrupt Wnt signalling and reduce osteoblastic bone formation.	(Gong *et al*. 2001)
	Osteogenesis imperfecta	Increased bone fragility; blue sclerae in some types	*COL1A1*, *COL1A2*, *CRTAP*, *LEPRE*, *PPIB*	Loss-of-function mutations in collagen and collagen- processing proteins cause abnormal osteoid matrix, thereby impairing normal bone formation.	(Baldridge *et al*. 2008; Sykes *et al*. 1986; van Dijk *et al*. 2009)
	Juvenile-onset Paget disease	Short stature, fractures, skull enlargement, progressive deafness	*TNFRSF11B* (*OPG*)	Loss-of-function mutations disrupt inhibition of RANKL by osteoprotegerin, causing increased osteoclastic resorption of bone.	(Chong *et al*. 2003)
	X-linked osteoporosis	Juvenile-onset fractures in males	*PLS3*	Loss-of-function mutations affect Plastin-3, an actin-binding protein. Mechanism osteoporosis is unknown.	(van Dijk *et al*. 2013)
Increased bone mass	Osteopetrosis	Increased bone mass with fractures	*TNFRSF11A (RANK), TNFSF11 (RANKL), CLCN7, TCIRG1, OSTM1*	Loss-of-function mutations affecting osteoclast differentiation and function cause reduced bone resorption.	(Frattini *et al*. 2000; Guerrini *et al*. 2008; Kornak *et al*. 2001; Pangrazio *et al*. 2006; Sobacchi *et al*. 2007)
	Sclerosteosis and Van Buchem disease	Increased bone mass, syndactyly, entrapment neuropathies	*SOST*	Loss-of-function mutations affect inhibition of Wnt signalling by sclerostin, causing increased osteoblastic bone formation.	(Balemans *et al*. 2001)
	Autosomal dominant high bone mass	Increased bone density, entrapment neuropathies, square jaw and torus palatinus	*LRP5*	Gain-of-function mutation in Wnt co-receptor causes increased osteoblastic bone formation.	(Boyden *et al*. 2002)

The canonical Wnt/β-catenin signalling pathway is the key regulator of osteoblasts, which mediate bone formation (Balemans *et al*. 2001, Gong *et al*. 2001, Boyden *et al*. 2002, Little *et al*. 2002, Glass *et al*. 2005). The major Wnt antagonist sclerostin (*SOST*) was first identified by studying subjects with high bone mass due to sclerosteosis and Van Buchem disease (Balemans *et al*. 2001, Brunkow *et al*. 2001). The importance of Wnt signalling was further highlighted by the discovery of activating and inactivating mutations of the Wnt co-receptor *LRP5*, which results in high and low bone mass, respectively (Johnson *et al*. 1997, Little *et al*. 2002).

The RANK-RANKL-OPG pathway regulates osteoclasts, which mediate bone resorption. This signalling pathway was discovered in a functional screen of tumour necrosis factor (TNF)/TNF receptor superfamily members. OPG is an endogenous inhibiting decoy receptor of RANKL related to the TNF receptor. *In vivo* overexpression of OPG in mice was found to cause osteopetrosis due to impairment of the later stages of osteoclast differentiation (Simonet *et al*. 1997). Subsequently, human osteopetrosis phenotypes with increased BMD were found to be caused by mutations of RANK, RANKL and other related genes involved in osteoclast differentiation (Coudert *et al*. 2015).

### Regulatory mechanisms in bone turnover

**Figure 1:**
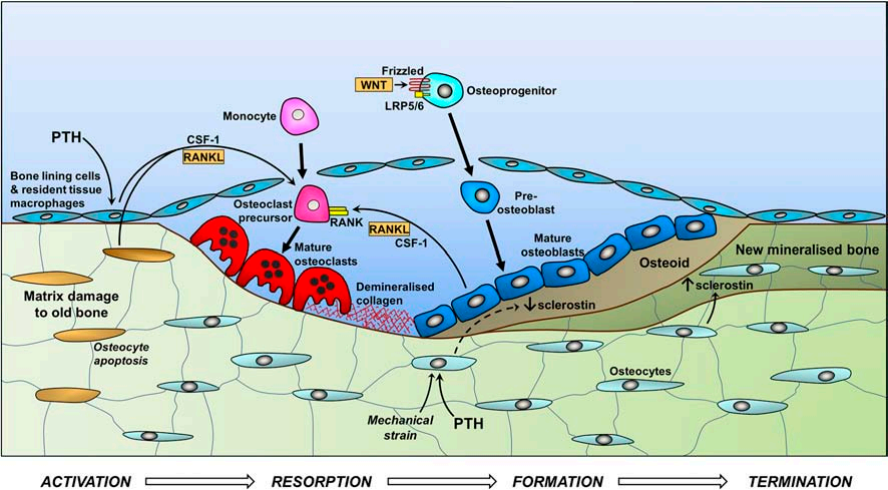
Schematic representing the bone remodelling processes of the ‘basic multicellular unit’ in the endosteal surface of trabecular bone. Activation: microdamage to the bone causes osteocyte apoptosis, reducing local basal inhibition of osteoclastogenesis. Resorption: in response to PTH signalling, RANKL and CSF-1 (colony-stimulating factor-1) increase recruitment, proliferation and differentiation of osteoclasts, which demineralise the bone matrix and then digest the collagen matrix, the remnants being removed by macrophages. Formation: PTH and mechanical activation of osteocytes reduce sclerostin expression, removing the potent inhibition of Wnt-mediated osteoblast differentiation (via cell surface receptor Frizzled and co-receptors LRP5 and LRP6) and bone formation. Termination: in response to increasing levels of sclerostin, bone formation ceases and newly deposited osteoid is mineralised.

Knowledge of the signalling pathways that regulate bone turnover is essential for understanding the pathophysiology of osteoporosis. The manifest complexity of the signalling pathways and networks that regulate the cellular processes involved in dynamic bone turnover is significant as small differences in function of individual components, including those not yet discovered, may have a combined effect on heritable risk of osteoporosis. The opposing processes of bone resorption and formation are tightly regulated by critical mechanisms including the Wnt signalling and RANKL/RANK/OPG pathways (Fig. 1). At the cellular level, bone remodelling takes place in multicellular units, which comprise co-located osteoclasts and osteoblasts within a bone remodelling cavity (Raggatt & Partridge 2010). The bone remodelling cycle is initiated by osteocytes in response to altered mechanical loading (Nakashima *et al*. 2011), local microdamage and systemic factors such as parathyroid hormone (Goldring 2015). Unloading stimulates expression of RANKL and the Wnt inhibitors sclerostin and Dickkopf-related protein 1 (DKK-1) in osteocytes, thus increasing osteoclastic bone resorption and decreasing osteoblastic bone formation. Repetitive loading can cause fatigue-induced microdamage and focal tissue injury that leads to osteocyte apoptosis, following which pro-osteoclastogenic signalling is initiated by a discrete population of adjacent osteocytes (Kennedy *et al*. 2012). By contrast, increased mechanical loading decreases expression of sclerostin and DKK-1 in osteocytes and leads to increased osteoblastic bone formation (Robling *et al*. 2008). In their basal state, osteocytes maintain quiescence by inhibiting osteoclastogenesis by secreting transforming growth factor β (TGFβ) and inhibiting Wnt-activated osteoblastic bone formation by secreting sclerostin (Heino *et al*. 2002, Tu *et al*. 2012). If TGFβ levels fall, bone-lining cells become activated and join osteocytes in secreting the cytokines monocyte/macrophage colony-stimulating factor (M-CSF) and RANKL that stimulate recruitment and differentiation of circulating osteoclast monocyte progenitors (Nakashima *et al*. 2011). Mature multi-nucleated bone-resorbing osteoclasts adhere to the cell surface and create resorption pits in the middle of the multicellular unit. The osteoclasts secrete acid and proteases including cathepsin-K that degrade the bone matrix, leaving demineralised collagen that is resorbed by macrophages. Wnt-activated osteoblasts subsequently secrete and mineralise new bone matrix (osteoid) to fill the resorption cavity. When the repair is complete, the bone surface returns to its quiescent state and sclerostin stimulates mineralisation of the new osteoid (Tu *et al*. 2012).

### Gene discovery drives therapeutic innovation

Identification of the genes and regulatory networks that determine normal bone formation, maintenance and strength has facilitated the recent development of new targeted treatments to increase BMD and reduce fracture risk. There is a pressing need for new treatment options in osteoporosis as current treatments achieve at best partial relative risk reduction of fracture. Bisphosphonates remain the mainstay of treatment for primary and secondary prevention of osteoporotic fracture, despite the recognition uncommon but significant side effects (Kennel & Drake 2009). Other treatments including strontium ranelate and raloxifene are no longer advocated as first line because of associated cardiovascular risks (Barrett-Connor *et al*. 2006, Abrahamsen *et al*. 2014).

New treatments for osteoporosis include small-molecule inhibitors and biological therapies have been developed based on this new understanding of bone turnover. Denosumab is a fully humanised monoclonal antibody to RANKL that mimics the endogenous inhibiting activity of OPG (Lacey *et al*. 2012). Denosumab is administered by 6-month injection; yet, its use is restricted by high cost and its effects are rapidly reversible. Another new class of osteoporosis drugs are those that target the osteoclast-secreted protease cathepsin-K (Makras *et al*. 2015). However, most of the cathepsin-K inhibitors have adverse off-target effects, with the exception of odanacatib, which has now successfully completed phase III trials (Bone *et al*. 2015). Humanised monoclonal antibodies to the Wnt antagonist sclerostin (romosozumab and blosozumab) are in development as new anabolic agents (Shah *et al*. 2015, Sim & Ebeling 2015). However, since romosozumab is currently in phase III trials and blosozumab has only completed phase II trials, effect on fracture risk is still unknown.

Despite some recent advances, there remains a large unmet need for new therapeutic targets in osteoporosis. Of all the newer targeted treatments, only the small-molecule odanacatib is administered orally. The requirement for parenteral subcutaneous administration is an impediment to use of teriparatide and all monoclonal antibody treatments. It is hoped that further advances in knowledge of the complex mechanisms of bone turnover regulation and the genetic basis of the heritability of osteoporosis will enable the accelerated development of new osteoporosis treatments. The current goals in osteoporosis gene discovery must be to identify novel targets with a systematic methodology that may replicate the successes of past studies in Mendelian extreme phenotype cohorts.

## Systematic osteoporosis gene discovery in human studies

### The Human Genome Project and association studies

In order to identify novel genes that relate to heritability of osteoporosis systematically, new population-based methodologies were required and have been used extensively. Linkage analysis and positional cloning techniques, used to identify gene mutations in Mendelian disorders, were less successful when applied to population cohorts with low BMD. Several genome-wide linkage studies were performed on high and low BMD cohorts to detect quantitative trait loci (QTLs) that regulate BMD. However, these studies produced heterogeneous results, and when combined in a meta-analysis, none of the QTLs reached genome-wide significance (Ioannidis *et al*. 2007).

It was the subsequent development of GWAS analysis that made it possible to search for genetic influences on complex polygenic traits such as osteoporosis. After the first sequence of the human genome was completed, the International HapMap Project was established to catalogue the common genetic variation between the genomes of 249 individuals in four different populations, leading to the mapping of over 1 million single-nucleotide polymorphisms (SNPs) within the human genome (International HapMap Consortium 2005). A major aim of the HapMap Project was to facilitate the study of the contribution of normal genetic variation to the inheritance of complex polygenic phenotypic traits such as osteoporosis (Frazer *et al*. 2009). In GWAS analyses, large population-based cohorts containing many thousands of subjects are genotyped by chip-based microarrays for the presence of millions of SNPs, or alternatively copy-number variants (CNVs). These data are used to perform hypothesis-free significance testing for association of the inheritance of the SNPs or CNVs with disease phenotypes (Manolio 2010).

To date, GWAS have identified more than 60 loci associated with BMD, confirming the polygenic nature of this variable (Richards *et al*. 2012). Less data are, however, available for fracture risk than BMD (Mitchell & Streeten 2013). Although estimation of areal BMD by DEXA scanning is a good predictor of fracture susceptibility, it does not necessarily reflect bone quality as DEXA does not account for bone geometry, size and strength (Marshall *et al*. 1996).

Population-based genetic cohort studies have significant limitations in relation to osteoporosis gene discovery. When GWAS data are combined in meta-analyses, variability in the characterisation of phenotype can be an important confounder, and population studies are also limited by the quality and reproducibility of the phenotyping (Visscher *et al*. 2012). Many of the genomic loci identified by GWAS map to genes in pathways already known to be related to bone biology, such as the Wnt/β-catenin, RANK-RANKL-OPG, mesenchymal stem cell differentiation and SOX9-regulated endochondral ossification pathways (Mitchell & Streeten 2013). Overall, GWAS analysis has not yet led to the discovery of major new mechanisms that regulate bone turnover regulation and predispose to osteoporosis. Indeed, contrary to initial expectations, GWAS analyses failed to discover sufficient determinants of polygenic heritable traits to enable individual disease risk prediction. An osteoporosis risk prediction model using the combined weighted effects of 63 BMD-decreasing alleles in a population-based study containing 2836 women explained only 5.8% of the total variance in femoral neck BMD (Estrada *et al*. 2012).

A further difficulty in identifying susceptibility genes by GWAS analysis arises from the fact that identified SNPs are rarely functionally significant, although their loci may identify adjacent pathogenic pathways and mechanisms. The co-association of the SNP with the disease phenotype indicates that it is in linkage disequilibrium with a gene (or a non-coding regulatory element) that has a constituent functional role that is contributory to the phenotype (Cantor *et al*. 2010). The size of the detected effect is itself of little importance, and it is typical that the effect size of individual identified loci is inversely proportional to the population sample size in a GWAS. The mean effect size of SNPs identified in the largest osteoporosis GWAS meta-analysis to date was 0.048 standard deviations (s.d.s) and the largest effect size was 0.1 s.d. (Estrada *et al*. 2012, Zheng *et al*. 2015).

To address the limitation that standard GWAS microarrays only test common genetic variants (minor allele frequency (MAF) > 5%), larger reference panels have been created from the recent UK10K and 1000 Genomes projects. ‘Next-generation sequencing’ techniques have enabled direct imputation of low-frequency coding and non-coding variants found by whole-genome sequencing of large osteoporosis cohorts. Thus far, only one low-frequency variant with genome-wide significance for skeletal disease has been identified by these techniques (Zheng *et al*. 2015). The variant is near a novel locus, engrailed homeobox-1 (*EN1*) and has an estimated effect size on lumbar spine BMD of 0.2 s.d.s, which is four-fold larger than the mean effect size of previously reported common variants.

### Transcriptomics and osteoporosis

One limitation of the GWAS data is that the majority of identified SNPs may be located within poorly annotated regulatory elements in intronic or intergenic non-coding regions (Cooper 2010). Indeed, it is increasingly appreciated that heritable variation of osteoporosis risk is manifest in more ways than just variation in the coding sequence of genes. Expression levels of RNA and proteins, as regulated by gene regulation networks and epigenetic control, are also of great importance. Consequently, study of variations in levels of RNA transcripts has also been applied to identify new molecular mechanisms and signalling pathways involved in regulation of BMD. Furthermore, it is anticipated that analysis of non-coding RNAs will lead to a better understanding of the significance of intergenic loci identified by GWAS (Hangauer *et al*. 2013).

Transcriptomic studies have used high-throughput microarray analysis to quantify mRNA expression in osteoporotic and non-osteoporotic human bone, precursor mesenchymal stem cells and primary osteoblast cultures (Wu *et al*. 2013). In a microarray study of gene expression in human osteoblasts, 1606 genes were found to be differentially expressed between osteoporotic and non-osteoporotic subjects (Trost *et al*. 2010). Many of these genes had been identified previously by GWAS analysis, but several were new and could not have been predicted. Thus, osteoporosis transcriptomics studies have confirmed that numerous distinct signalling pathways are involved in the regulation of bone turnover and bone mass. However, replication of these studies has been difficult and confounded by limited sample size, poor signal to noise ratio and technical limitations of commercially available microarrays (Wu *et al*. 2013). Future work will increasingly use next-generation sequencing methods, which do not require *a priori* knowledge of the transcriptome sequence and have a superior sensitivity, specificity and dynamic range in comparison with current microarrays (Vikman *et al*. 2014). With use of advanced statistical techniques, it is possible to discover new splice variants and non-coding sequences. Despite these advances, RNA-sequencing approaches require preparation of high-quality RNA from sufficient numbers of physiologically relevant samples. Accordingly, it is not yet possible to study gene expression in osteoporosis using large population-based sample sizes, and RNA sequencing is likely to be most useful in the analysis of animal models.

### Proteomics

Proteomics allows the simultaneous analysis of all proteins in a sample of cells by antibody-based purification and mass spectrometry techniques. A number of studies have demonstrated differential regulation of cellular protein expression in osteoporosis (Wu *et al*. 2013). Most of the studies have been performed *in vitro* using osteoblast and osteoclast cell cultures. Human proteomic studies have predominantly used peripheral circulating monocytes as precursors to osteoclasts (Deng *et al*. 2011*a*,*b*), or bone marrow-derived mesenchymal stem cells (Choi *et al*. 2010). However, reproducibility of such results has been limited.

### Epigenomics

The heritability of osteoporosis may also be mediated by epigenetic gene regulation, in which gene or allele expression is ‘imprinted’ by DNA methylation and histone modifications (Holroyd *et al*. 2012). Epigenome-wide studies have only recently been performed in relation to osteoporosis. Transcriptome gene expression microarray, epigenomic miRNA microarray and methylome sequencing were simultaneously performed using circulating monocytes from five subjects with low hip BMD and five with high BMD, in order to integrate transcriptomic and epigenomic data (Zhang *et al*. 2015). The aim is to reveal the higher regulatory mechanisms (‘interaction network modules’) underlying the genetic control of osteoporosis heritability. However, the problems of low sample number and difficulty of accessing human skeletal tissues are important limitations in such approaches.

## The mouse as an essential tool for osteoporosis gene discovery

Human population studies have been limited in their ability to identify novel genetic determinants of osteoporosis, although they have confirmed that bone-regulating genes identified in the study of rare extreme phenotypes contribute to the complex heritability of secondary osteoporosis. Across the breadth of human biology, there are huge knowledge gaps as the function of most genes and the heritability of many complex diseases remain unknown (Schofield *et al*. 2012). In order to meet the challenge of discovering the functional relationship between human genes and their phenotypic effects, powerful new experimental tools are required. Model organisms provide the ability to draw genetic and physiological parallels to human genetic systems, and their use has complemented many advances in human molecular genetics. In particular, an extensive range of techniques for experimental genetic manipulation have been developed in mice.

The genome of the C57BL/6J mouse strain was sequenced in 2002 (Waterston *et al*. 2002), shortly after the human genome. Thus, laboratory mice provide a unique resource that can be used to generate genetically modified models of human diseases (Schofield *et al*. 2012). Currently, 17,055 mouse–human homologs have been annotated in the Mouse Genome Database (Dolan *et al*. 2015). Critically, the selective mutation or deletion (‘knockout’) of individual genes in mice can be used to identify and characterise gene function *in vivo*.

### International knockout mouse programmes

Such is the utility of knockout mice that a number of mutagenesis programmes have collaborated to cryo-preserve mouse lines for unrestricted use by the research community. The IKMC was established with the goal of creating a complete resource of reporter-tagged null mutations for all protein-coding genes in C57BL/6 mouse ES cells by 2021 (Collins *et al*. 2007).

**Figure 2:**
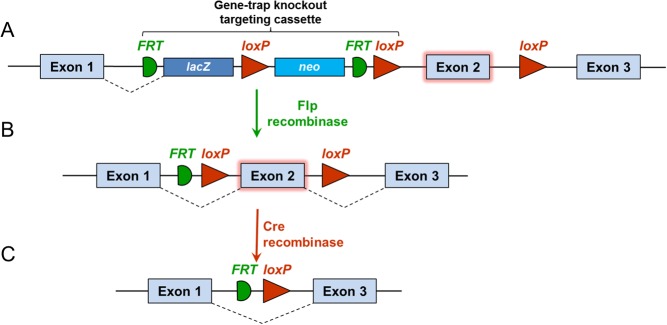
Knockout-first strategy for creating dual-purpose knockout/conditional alleles. Bacterial artificial chromosome (BAC)-based targeting vectors are inserted by homologous recombination into mouse ES cells. Recombination steps with Cre or Flp recombinase are illustrated. (A) Knockout-first allele (reporter-tagged insertion allele). Gene-trap knockout is generated using a targeting cassette containing the marker genes lacZ and neomycin. A separate *loxP* site is inserted on the other side of a critical exon (Exon 2). (B) Conditional allele (post-Flp). By crossing mice with a Flp deleter strain, the gene-trap knockout is reversed and a floxed allele is created, enabling conditional Cre recombinase-mediated gene inactivation. (C) Deletion allele (post-Flp and Cre with no reporter).

The IKMC-targeted mutagenesis techniques involve use of bacterial artificial chromosome (BAC)-based ‘Knockout-First’ conditional ready gene targeting vectors that use homologous recombination to incorporate a lacZ selection cassette in the relevant targeted sequence (Fig. 2) (Testa *et al*. 2004). This enables the generated mouse lines to be versatile and powerful tools for research. The Knockout-First vectors combine two functions by creating either a reporter-tagged knockout or a conditional mutation if the gene-trap cassette is removed by FLP recombinase, thereby reverting the knockout mutation to wild type, although with addition of *loxP* sites that flank a functionally critical exon (Skarnes *et al*. 2011). The consequence of this is that temporal or tissue-specific analysis of gene function can be performed by crossing mice bearing the allele containing the *LoxP*-flanked (‘floxed’) critical exon with transgenic mice that express Cre recombinase under control of a constitutive or inducible cell type-specific promoter.

### Pipeline phenotyping of knockout mice

The IKMC provides readily available knockout mice that can be used for functional investigation of candidate genes, for example in loci identified by GWAS analysis (Cox & Church 2011). The singular ambition to deduce the function of all the genes discovered in the Human Genome Project, however, has led to the development of ambitious large-scale systematic phenotyping programmes of all knockout mouse lines with deletions of homologs to human genes (Bradley *et al*. 2012). These projects enable the study of the extreme phenotype potential of every known coding gene, thereby providing the potential for systematic discovery of novel critical skeletal genes. What would otherwise have been individually impossible in terms of resources and complexity has been achieved by the coordination and the standardisation of a series of international programmes to form a major worldwide project The IMPC (Koscielny *et al*. 2014).

The international phenotyping pipeline incorporates standardised and validated tests with protocols shared by contributing mouse clinics across the world (de Angelis *et al*. 2015). The pipelines incorporate 20 phenotyping tests that capture 413 parameters and cover all systems divided between the categories of morphology, metabolism, cardiovascular, bone, neurobehavioural and sensory, haematology, clinical chemistry and allergy/immune. The phenotyping tests are performed at defined times in the first 16 postnatal weeks and require a minimum cohort of seven male and seven female mice (Brown & Moore 2012). New statistical models have had to be developed, in order to ensure the reproducibility of the multivariate data generated (Karp *et al*. 2015). In an initial report of phenotype data on mutant lines representing 320 unique genes, 83% of mutant lines were outliers (de Angelis *et al*. 2015). Of the 250 lines reported in an early phase of the phenotyping pipeline contributed by the IMPC member Wellcome Trust Sanger Institute (WTSI), 104 were lethal or sub-viable and were phenotyped as heterozygotes; nonetheless, haploinsufficient phenotypes were detected in 38 of these lines (White *et al*. 2013). Ongoing results from the international multicentre phenotyping projects are released at http://www.Mousephenotype.org/.

## Skeletal phenotyping screen of knockout mouse lines

### Ancillary IMPC projects

The systematic phenotyping of knockout mice by the IMPC has already begun to reveal genes whose deletion has an effect on BMD (estimated by whole-body DEXA), implying a functional role in bone development and skeletal physiology. To date, 79 out of 1820 lines have an outlier phenotype associated with decreased BMD (IMPC phenotype MP:0000063) and 41 lines have increased BMD (IMPC phenotype MP:0000062). However, a major limitation is the poor sensitivity and specificity of DXA for the analysis of the mouse skeleton (Holmen *et al*. 2004). Other skeletal parameters included in the IMPC phenotyping are body length and X-ray skeletal survey to detect gross anatomical variation. As critical determinants of healthy bone include bone mineral content (BMC), bone strength and other three-dimensional morphological parameters, the IMPC phenotype screen lacks the precision required for comprehensive detection of bone structure and strength abnormalities.

**Figure 3:**
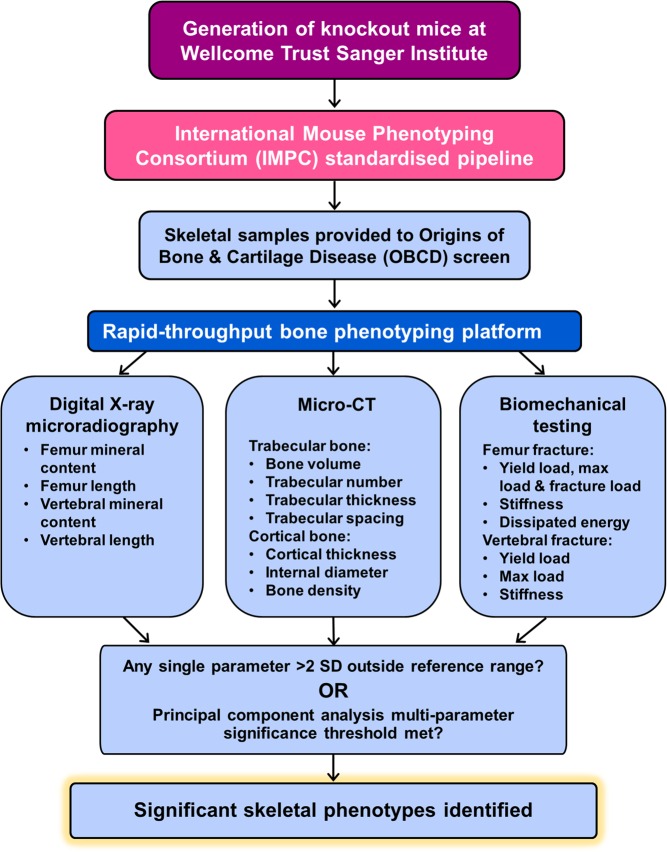
Flow chart showing how the OBCD bone phenotyping platform leads to identification of significant abnormal skeletal phenotypes, in conjunction with the IMPC standardised phenotyping project.

To address these limitations, a number of ancillary specialist screens have been established. The Origins of Bone and Cartilage Disease (OBCD) project (http://www.boneandcartilage.com/) is performing a multi-parameter skeletal phenotyping screen of mouse lines generated by the WTSI, an IMPC partner institution, using methods that give critical functional and structural information regarding skeletal phenotype (Fig. 3). The goals of the OBCD project are to (i) identify novel pathways regulating normal bone development, maintenance and resilience; (ii) uncover new genetic determinants of osteoporosis; and (iii) provide *in vivo* models to elucidate their molecular basis and investigate novel treatments.

**Figure 4:**
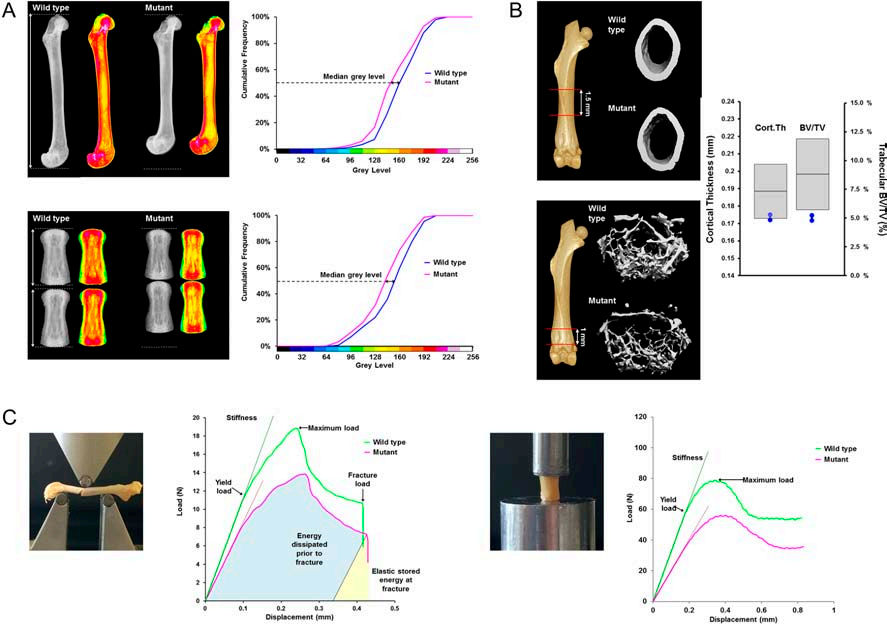
OBCD skeletal phenotyping methods. (A) X-ray microradiography images of femur and fifth to sixth tail vertebrae from wild-type and mutant mice. Low bone mineral content is represented in green/yellow colour and high bone mineral content is represented in red/pink colour in pseudo-coloured images. Cumulative frequency graphs showing difference in bone mineral content between wild-type and mutant mice. (B) Micro-CT images of cortical and trabecular bone from wild-type and mutant mice. Cortical thickness and trabecular bone volume/total volume (BV/TV) parameters in the mutant are shown in comparison with reference mean ± 2 standard deviations. (C) Femur three-point bend and vertebral compression analysis with load–displacement curves illustrating biomechanical parameters.

New imaging and biomechanical techniques have been developed to detect abnormalities of bone structure and strength that parallel those occurring in human disease. Cross-disciplinary collaboration with the fields of biophysics, microimaging and statistics has enabled development of a bespoke rapid-throughput multi-parameter bone phenotyping platform (Fig. 4) (Bassett *et al*. 2012*a*, Esapa *et al*. 2012).

### OBCD phenotyping techniques

Limbs and caudal vertebrae from knockout mice are analysed at 16 weeks of age after completion of the IMPC phenotyping pipeline at WTSI. No additional animals are required for the OBCD screen, and transportation of samples is logistically simple. Each batch of 75 samples, which contains both mutants and wild-type controls, is analysed blind with genotypes only being assigned on completion of the batch’s phenotyping.

Digital X-ray microradiography is performed on femurs and tail vertebrae, and the images are analysed to determine bone length and BMC (Bassett *et al*. 2012*b*). X-ray images are recorded at 10 µm pixel resolution using a Faxitron MX20 specimen radiography system. Bone length is determined using ImageJ 1.41 software (http://rsb.info.nih.gov/ij/). The relative mineral content of the calcified tissues is quantified after calibrating each image to three internal standards. Measurement of the median grey level is used to identify outliers with increased or decreased BMC relative to reference data obtained for more than 100 wild-type controls of the same genetic background.

Micro-CT analysis has been optimised to determine femoral trabecular bone parameters including trabecular bone volume as proportion of tissue volume (BV/TV), trabecular thickness and trabecular number. In addition, cortical bone parameters of cortical thickness, cortical diameter and cortical volumetric BMD are calculated (Bouxsein *et al*. 2010, Esapa *et al*. 2012). A Scanco micro-CT 50 system allows automated rapid-throughput high-resolution imaging. Three-dimensional quantitative image analysis is performed using clearly defined regions of interest and compared with reference data.

Biomechanical variables of bone strength and toughness are derived from femur three-point bend test load–displacement curves and tail vertebrae compression testing (Esapa *et al*. 2012). Load–displacement curves are plotted so that yield load, maximum load and fracture load can be determined. Stiffness is determined from the slope of the linear (elastic) part of the load–displacement curve.

### Pilot study

Before commencing the OBCD skeletal phenotyping screen, a prospective pilot study of 100 unselected knockout mouse lines was undertaken (Bassett *et al*. 2012*a*). As part of the pilot project, it was necessary to determine reference ranges and coefficients of variation for each of the study parameters for female C57BL/6 wild-type mice. Principal component analysis was used to optimise the detection of significant skeletal phenotypes in multivariate outliers, as significant abnormal pheno­types may only be detected when variances in all para­meters are considered (Rousseeuw & Van Zomeren 1990). As reference data were established in a large number of genetically identical wild-type mice, power calculations determined that samples from only two animals were required to detect a significant abnormality that represents an outlier phenotype.

In the pilot study of 100 knockout mouse lines, nine new genetic determinants of bone mass and strength were identified, none of which had been identified previously in GWAS analysis of osteoporosis cohorts or could have been predicted *a priori*. Analysis of the contrasting patterns of abnormality amongst the different phenotypic parameters enabled phenotypes to be categorised in a way that could be mapped directly to human skeletal disease. Bones were classified as either (i) weak but flexible with low BMC (as in osteoporosis), (ii) weak and brittle with low BMC (typical of matrix disorders including osteogenesis imperfecta) or (iii) strong but brittle with high BMC (high bone mass disorders such as osteopetrosis). The nine new determinants of bone mass and strength identified included five genes whose deletion results in low bone mass (*Bbx*, *Cadm1*, *Fam73b*, *Prpsap2* and *Slc38a10*) and four whose deletion results in high bone mass (*Asxl1*, *Setdb1*, *Spns2* and *Trim45*). The study also confirmed the low bone mass phenotype identified previously in *Sparc* knockout mice (Delany *et al*. 2000). Three of the knockout lines carried heterozygous mutations (*Asxl1*, *Setdb1* and *Trim45*), whereas the rest were homozygotes.

### Other skeletal phenotyping programmes

Although the OBCD pilot study was the first approach to be published, similar phenotype screening methods have been undertaken by others. Lexicon Pharmaceuticals, Inc. recently published selected results from a screen of knockout mouse lines to search for potential osteoporosis drug targets (Brommage *et al*. 2014). This phenotyping screen included three techniques (skeletal DEXA of live mice, micro-CT of dissected bones and histological examination of decalcified bones). Ten novel genes were named, and three further unnamed novel genes coding for apparent potential osteoporosis drug targets were alluded to.

The IMPC-constituent knockout mouse programme (KOMP) of the Jackson Laboratory has recently commenced its own skeletal phenotyping project that involves rapid micro-CT and automated bone and joint cartilage histology (http://bonebase.org/">http://bonebase.org/">http://bonebase.org/). This screen focuses on detecting evidence of variations in skeletal cellular function. Histomorphometry is performed by a recently innovated high-throughput process that involves computer-automated signal detection for the particular cell type-specific stains. Data are accrued by automated analysis that calculates the percentage of the bone surface containing the light signal from each stain, thereby suggesting the pattern of disruption of cellular activity in the trabecular bone of the femur and vertebra that may account for the architectural observations seen in micro-CT (Hong *et al*. 2012). Besides phenotyping inbred lines from the IKMC mutant mouse repository (Yoshiki & Moriwaki 2006), the Bonebase phenotyping project is phenotyping mouse lines from the ‘Collaborative Cross’ project, which has created hybrids from eight founder inbred strains in order to perform genetic mapping studies to identify the QTLs that contribute to complex traits and diseases (Bogue *et al*. 2015). Similarly, Bonebase is also studying ‘diversity outbred’ lines created to produce a genetic resource to facilitate high-resolution mapping of the effects of allelic heterozygosity that replicates the complexity of the human population (Svenson *et al*. 2012).

### Current OBCD project goals

The OBCD project is currently funded by a Wellcome Trust Strategic Award to undertake skeletal phenotyping of all knockout mouse lines generated at the Sanger Institute. Results are available at the OBCD website and also uploaded to the IMPC mouse portal. Although the IMPC parent project is powered robustly to assess and catalogue the unknown pleiotropic effects of gene deletion, the OBCD screen is designed for rapid-throughput hypothesis generation. Once extreme phenotypes are detected, they can be selected for additional in-depth analysis.

### Detailed analysis of extreme phenotypes

**Figure 5:**
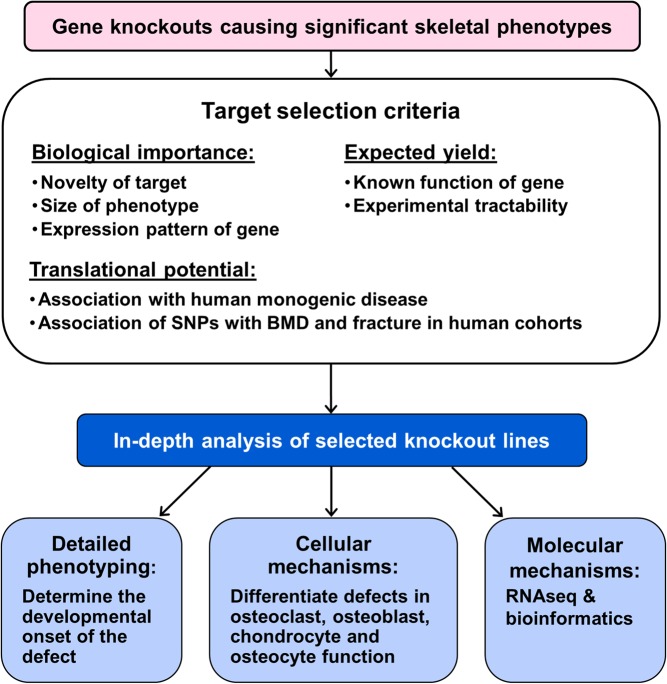
Flow chart outlining selection of knockout mouse lines for further study and analysis.

Knockout mice with extreme skeletal phenotypes are considered for additional detailed analysis and the selection procedure follows a specific algorithm (Fig. 5). Although novelty is a key criterion, phenotype severity, biological plausibility, human disease association and experimental tractability are also critical considerations (Duncan *et al*. 2011, van Dijk *et al*. 2014).

Detailed phenotyping includes skeletal analysis during prenatal and postnatal development, at peak bone mass and in adulthood. Juvenile analysis establishes the role of gene in skeletal development and growth, whereas adult analysis establishes its role in skeletal maintenance and repair. Gene expression pattern of the deleted gene can be investigated by LacZ staining, taking advantage of the reporter gene included in the knockout-first gene targeting cassette. Temporal expression can be established at different time points in embryonic and adult mice.

To determine the cellular basis of the abnormal phenotype, static and dynamic histomorphometry can be performed to identify abnormalities of osteoclastic bone resorption and osteoblastic bone formation (Bassett *et al*. 2010). Primary chondrocytes, osteoblasts and osteoclasts, cultures from wild-type and mutant mice can be undertaken to determine the consequences of gene deletion on cell proliferation, differentiation and function (Bassett *et al*. 2008). If these studies suggest that the phenotype is a consequence of a defect in a specific bone cell lineage, conditional deletion of the gene in the specific cell type may be used to determine if the phenotype of the global knockout is recapitulated. By crossing the knockout mouse line with an Flp deleter strain, the gene-trap knockout is reversed and a *loxP*-flanked (‘floxed’) allele is generated (Fig. 2). Subsequently, floxed mice can be crossed with bone cell lineage-specific Cre strains (Murray *et al*. 2012) (Table 2).

**Table 2:** Examples of cell-specific promoter-driven Cre recombinases in available transgenic mouse lines

**Cell type**	**Mouse lines expressing Cre-recombinase with cell-specific promoter**	**Equivalent inducible Cre-recombinase**	**References**
Osteoblasts	*OC*-Cre, *Col1a1*-Cre	*Col1*-CreER^T2^	(Kim *et al*. 2004; Nakanishi *et al*. 2008; Zhang *et al*. 2002)
Osteoclasts	*Ctsk*-Cre, *LysM*-Cre	*Ctsk*-CreER^T2^	(Chiu *et al*. 2004; Sanchez-Fernandez *et al*. 2012)
Osteocytes	*Dmp1*-Cre	*Dmp1*-CreER^T2^	(Lu *et al*. 2007; Powell *et al*. 2011)
Chondrocytes	*Col2*-Cre	*Agc1*-CreER^T2^	(Henry *et al*. 2009; Yoon *et al*. 2005)

To determine the molecular basis of the skeletal phenotype, the effect of the gene knockout on global gene expression can be examined by whole-genome microarray analysis of RNA extracted from bones of the knockout mice or from cell cultures. Cluster analysis is performed to identify patterns of gene expression that suggest involvement of specific signalling pathways. In addition, whole transcriptome analysis can be performed by RNA sequencing with next-generation sequencing techniques. Bioinformatics analysis can determine the effect of the gene deletion on complex gene networks including alternative splice variants and regulatory elements such as non-coding RNAs (Vikman *et al*. 2014).

Although the rapid-throughput skeletal phenotyping screen is a powerful technique to identify new gene that regulates bone mineralisation and strength, it has limitations related to (i) the use of mice as a model system, (ii) analysis of only knockout animals and (iii) the specific skeletal phenotyping methods selected.

There are well-described differences in bone structure, bone remodelling and hormonal changes between humans and mice. Despite these differences, key molecules that regulate bone and cartilage have been shown to have the same functions in mice and humans and many inherited skeletal disorders are recapitulated in genetically modified mice. In addition, although there is no mouse menopause, the consequences of oestrogen deficiency can still be studied in mice using the ovariectomy provocation model. Furthermore, if extreme skeletal phenotypes are identified in mouse knockout lines, genetic variation in the homologous human genes can be investigated in large well-characterised human populations such as those included in the GEnetic Factors for OSteoporosis Consortium (GeFOS) (http://www.gefos.org/).

The IKMC/IMPC knockout strategy aims to determine the physiological role of genes by identifying the pathophysiological consequences of loss-of-function but will not identify phenotypes associated with gain-of-function mutations, epigenetic modifications or other environmental risk factors. Nevertheless, if a significant skeletal phenotype is detected in knockout animals, the consequences of gain-of-function mutation could then be studied by using CRISPR-Cas-based targeted gene-editing techniques to generate mouse models (Gaj *et al*. 2013, Sander & Joung 2014).

Robust, sensitive and specific skeletal phenotyping screening requires a combination of complementary, rapid-throughput methodologies but can never be exhaustive. However, once an extreme phenotype has been identified and prioritised for further detailed analysis, other important determinants of bone strength such as tissue mineralisation and bone geometry can be determined by quantitative backscattered electron scanning electron microscopy (Bassett *et al*. 2010) and statistical shape modelling (Yang *et al*. 2006), respectively.

In conclusion, systematic, rapid-throughput skeletal phenotyping of genetically modified mice is an exciting new approach that complements human population studies of complex polygenic disorders and has the potential to identify important new signalling pathways involved in the pathogenesis of skeletal disease.

## Declaration of interest

The authors declare that there is no conflict of interest that could be perceived as prejudicing the impartiality of this review.

## Funding

This work was supported by a Wellcome Trust Strategic Award (grant number 101123) and a Wellcome Trust Project Grant (grant number 094134).
